# Facial skin metastasis of colorectal cancer: a case report

**DOI:** 10.1186/1757-1626-3-28

**Published:** 2010-01-15

**Authors:** Zilvinas Saladzinskas, Algimantas Tamelis, Saulius Paskauskas, Darius Pranys, Dainius Pavalkis

**Affiliations:** 1 Departments of Surgery, Kaunas University of Medicine, Kaunas, Eiveniu 2, 50009 Kaunas, Lithuania; 2 Department of Pathology, Kaunas University of Medicine, Kaunas, Eiveniu 2, 50009 Kaunas, Lithuania

## Abstract

**Introduction:**

Liver and lungs are common locations of distant metastases of colorectal cancer. Skin metastases of colorectal cancer are very rare, and facial lesions are extremely uncommon.

**Case presentation:**

An anterior resection of the rectum was performed for rectal cancer T3N0M0G3. A small ulcer on the upper lip developed 3.5 years after primary operation. Metastasis of adenocarcinoma was confirmed histologically, and local excision was performed. At the same time, a solitary metastasis in the right lung was diagnosed, and the right lower lobectomy was performed. No other metastasis or local recurrences were observed during the next 7 months.

**Conclusion:**

Skin metastases in the face from colorectal cancer are very rare and may indicate tumour relapse several years after primary resection. These patients have a worse prognosis.

## Introduction

Liver and lungs are common locations of distant metastases of colorectal cancer. Skin lesions are very rare they occur in 4-6.5% of cases [1,2]. The most frequent localization of the skin metastases is postoperative scars [3]. Facial lesions are extremely uncommon. Literature mentions only a few cases of such pathology. Overall, skin metastasis is a poor prognostic marker, and medium survival is very short [2]. We report the second case of the metastasis of the colorectal cancer to the lip.

## Case presentation

A circular tumor of the rectum located at 10 cm from the dentate line was diagnosed 4.5 years ago in a 65 year-old Lithuanian male. Histologically, well-differentiated adenocarcinoma G1 was identified. No distant metastases were found, and endorectal ultrasound revealed uT3N0 stage of the disease. Preoperative radiation was applied with the total dose of 45 Gy. After 6 weeks, low anterior resection with preventive ileostomy was performed. The postoperative period was uneventful. Postoperative histological examination showed infiltrative mucinous adenocarcinoma of the rectum pT3 N0 M0 L0 V0 R0 G3, Astler - Coller B1. Six postoperative courses of 5-fluorouracil were applied. Preventive ileostomy was closed uneventfully on the 7^th ^week after primary operation.

The follow-up included twice-yearly abdominal ultrasound, chest x-ray, colonoscopy, and carcinoembryonic antigen.

**Figure 1:**
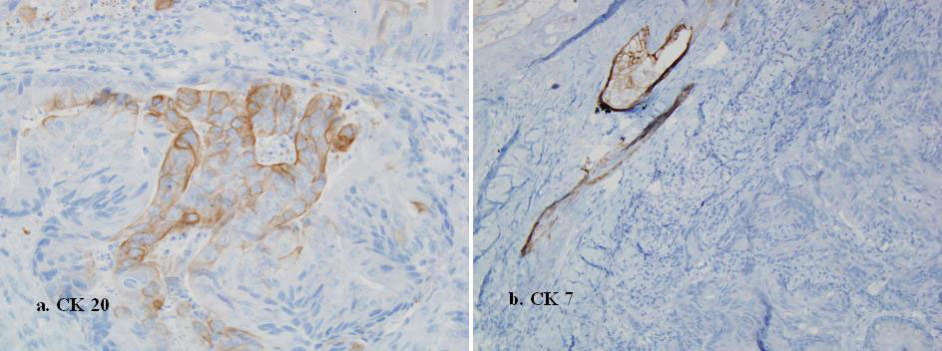
**Immunohistochemically, the carcinoma cells, were focally positive for Cytokeratin (CK) 20 (a), but negative for CK 7 (b)**. This CK staining pattern suggested that the skin tumor was a metastasis from the previously resected rectal cancer.

A small 0.5 cm diameter ulcer on the upper lip developed after 42 months following the removal of the primary tumor, and biopsy confirmed metastasis of the colorectal adenocarcinoma (Figure 1a. and 1b). This lesion was excised.

Simultaneously, chest computed tomography scan showed a solitary mass of 3.4 cm in diameter in the lower lobe of the right lung, irregularly accumulating contrast medium. Consequently, lobectomy was performed. Postoperative histological examination showed moderately differentiated metastasis of the colorectal cancer G2. The postoperative period was uneventful. No progress of the disease was observed during the subsequent 7 months.

## Discussion

We have found only 15 articles describing metastases of colorectal cancer to the skin of head and neck. Facial localization is especially rare; and it has been mentioned in only in the 4 publications in English [1,3-5]. One manuscript of these described facial metastasis involving oral commisure [5] and our manuscript report the second case of the metastasis of the colorectal cancer to the lip.

Cancer of the large bowel most frequently metastasizes into the liver and lungs, but a skin lesion is very rare and could be observed in only 4-6.5% of cases [2]. Skin deposits are more typical for already metastatic colorectal cancer [3]. Metastatic lesions of the skin occur more frequently in cases of primary tumors of the lungs (up to 28.6% of all metastases) and breasts with equal distribution between males and females [2].

The most frequent localization of skin metastases of the colorectal cancer is abdomen, especially in the postoperative scars - up to 0.6% of all patients [3]. According to the data of one of the largest reviews of colorectal skin metastases, in 3 (3.9%) out of 77 patients they occurred at the site of postoperative scar [2]. On the average, skin metastases occur after 4.9 years following the excision of the primary tumor.

Clinically, metastases present as nodules, ulcers, cellulites or fibrous depositions. Histologically, they are classified as adenocarcinoma, squamous carcinoma, non-differentiated carcinoma, or other types. All described facial metastases from colorectal cancer were adenocarcinomas.

Colonic mucosa typically expresses cytokeratin (CK) 20 but not CK7. In this case CK20+/CK7-profile has been used to distinguish colonic adenocarcinoma from others arising in the lung, breast, or genitourinary tract, salivary gland. CK7 expression in colorectal adenocarcinoma has been reported to be rare, and when present, a metastatic origin needs to be excluded.

Skin metastases are a poor prognostic sign and medium survival after diagnosis ranges between 2 and 4.5 months [2]. Slightly longer survival more than 18 months is observed in cases of head and neck lesions [3].

Colon carcinoma is believed to metastasize initially through lymphatics and later through the haematogenous route. In this case, it is interesting and not clear, how after 42 months following the radical resection of the primary tumor (pT3 N0 M0 L0 V0 R0) and postoperative courses of the chemotherapy, the tumor metastasized to the facial region and lung.

## Conclusion

Skin metastases in the face from colorectal cancer are very rare and may indicate tumour relapse several years after primary resection. These patients have a worse prognosis.

## Abbreviations

CK: cytokeratin.

## Consent

Written informed consent was obtained from the patient for the publication of this case report. A copy of the written consent is available for review by the Editor-in-Chief of this journal.

## Competing interests

The authors declare that they have no competing interests.

## Authors' contributions

ZS operated the patient and performed an anterior resection of the rectum, later closed preventive ileostomy, and was a major contributor in writing the manuscript. AT was the doctor of the patient and observed him at ambulance, analyzed and interpreted the patient data regarding the metastatic large bowel cancer and contributed in writing the discussion of the manuscript. SP performed literature search and analysis regarding metastatic rectal cancer and metastatic skin tumors, analyzed and interpreted the patient data. DP performed the histological examination of the resected rectal cancer and excised skin lesion. DPS analyzed and interpreted the patient data and contributed in writing the discussion of the manuscript, critically revised it for important intellectual content. All authors gave final approval of the version to be published.
